# Biodegradable polysaccharide grafted polyacrylamide inhibitor for corrosion in CO_2_- saturated saline solution

**DOI:** 10.1016/j.heliyon.2023.e20304

**Published:** 2023-09-20

**Authors:** Rem Jalab, Mohammed Saad, Ahmed Benali, Ibnelwaleed A. Hussein, Mazen Khaled

**Affiliations:** a Gas Processing Center, College of Engineering, Qatar University, PO Box 2713, Doha, Qatar; b Chemical Engineering Department, College of Engineering, Qatar University, PO Box 2713, Doha, Qatar; c Department of Chemistry and Earth Sciences, College of Arts and Sciences, Qatar University, PO Box 2713, Doha, Qatar

**Keywords:** Carbohydrate polymers, Agar grafted polyacrylamide, CO_2_ corrosion, Carbon steel, Electrochemical measurements

## Abstract

A biodegradable polysaccharide-based inhibitor is grafted with polyacrylamide (PAM) for oilfields’ sweet corrosion. The green properties of agar and PAM were incorporated to synthesize an agar-grafted-PAM (AGGPAM) inhibitor. Electrochemical tests of Tafel and AC impedance, were used to determine the corrosion rate of carbon steel (C-steel) and protection efficiency in CO_2_-saturated 3.5 wt% NaCl solution. The surface morphology was characterized using FESEM coupled with EDX. Results demonstrated the promising performance of AGGPAM in improving steel resistivity, achieving 85% efficiency at 500 mg L^−1^ and reducing the corrosion rate from 33 to 4.9 mils per year at 25 °C. The electrochemical tests classified AGGPAM as a mixed-type inhibitor, yet with a larger potential to inhibit the cathodic hydrogen evolution. Kinetics study at a temperature of 50 °C revealed a deteriorated AGGPAM inhibition attributed to electrolyte diffusion through the weakly adsorbed AGGPAM film. Nevertheless, the AGGPAM-inhibited solution exhibited a corrosion rate of 26.7 mils per year at 50 °C, which is still lower than that of blank at 25 °C. The steel resistance was diminished from 1436 to 355 Ω cm^2^ at 50 °C. Implementing AGGPAM coating reduced the steel corrosion rate to 9.6 mils per year, achieving 71% efficiency. AGGPAM inhibitor toxicity was evaluated using ADMETlab, which predicted negligible hazardous impacts. Lastly, potentiostatic testing of steel with AGGPAM at an applied potential of 50 mV illustrated surface protection and decreased current over a prolonged time. Herein, the experimental investigation revealed the promising capabilities of AGGPAM as an efficient corrosion inhibitor in oilfields.

## Graphical abstract

**Figure:**
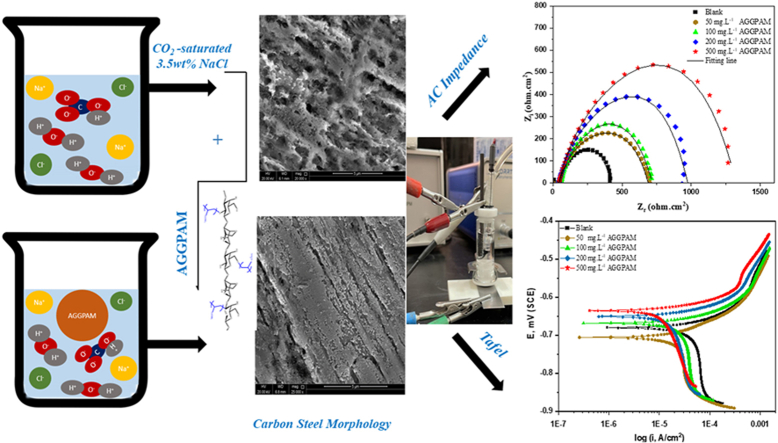

## 1 Introduction

The continuously increasing demand for fossil fuel energy with the lack of fast-developing clean energy technologies is raising the dependency on the oil and gas industry [1]. Inherently corrosive impurities of produced water, organic acids, and gases such as CO_2_ and H_2_S accompany sour crude oil and gas extraction. With the long-term operation of oil and gas transportation pipelines, the threats of corrosion attacks are highly encountered, especially at temperatures of 150 °C and depths of more than 3000 m [2]. Specifically, a CO_2_-rich environment alleviates sweet corrosion of the most utilized pipe materials of carbon steel (C-steel) and stainless steel [3]. C-steel is broadly used in the oil industry owing to its low price and effectiveness, given the high strength of its structural composition [4]. Yet, this steel type is of increased susceptibility to corrosion. The severity of corrosion caused by carbonic acid (H_2_CO_3_) formed during CO_2_ dissolution in water is considered to have more detrimental impacts than strong acids, leading to the disintegration of pipes' surface and internal walls [5,6]. For this reason, research attention has been intensively devoted to tackling pipeline corrosion, aiming to reduce maintenance costs and consequential shutdowns [7,8]. Despite the economic losses caused by the reduced well's productivity, the corrosion damages in the oil industry cost up to 170 billion USD annually [9].

Injection of corrosion inhibitors (CIs) is a convenient approach to overcome the hazardous effects without altering the conditions of exploration surrounding or interrupting the already-operating process in the oil and gas industry. These chemical compounds can be introduced to the pipelines of oil wells through the techniques of squeeze treatment, drip dosage, and capillary tube [10].

The inhibition mechanism commonly relies on the adsorption of injected molecules of chemical formulations onto the steel surface of pipes, composing an impeding film at the solid-liquid interface. Studies have documented the efficient role of organic chemicals containing nitrogen as CO_2_ corrosion inhibitors when injected internally in pipelines [[11], [12], [13], [14]]. A competent CI possesses electron donor sites owing to existing heteroatoms (N, O, S), π-electrons, and polar functional groups, which bind with the unoccupied orbitals of iron atoms of steel surface [15]. The reactions covering the mechanisms of corrosion attack in the Cl^−^ containing medium and adsorption of inhibitor onto the steel surface are described below in equations (1), (2), (3), (4), (5), (6), (7), (8), (9).

Metallic dissolution (anodic reactions):

(1) $$\begin{array}{c} Fe+{Cl}^{-}↔{FeCl}^{-} \end{array}$$

(2) $$\begin{array}{c} {FeCl}^{-}↔FeCl+e^{-} \end{array}$$

(3) $$\begin{array}{c} FeCl\to {FeCl}^{+}+e^{-} \end{array}$$

(4) $$\begin{array}{c} {FeCl}^{+}↔{Fe}^{2+}+{Cl}^{-} \end{array}$$

(5) $$\begin{array}{c} {FeCl}^{-}+Inhibitor^{+}↔FeCl^{-}..Inhibitor^{+} \end{array}$$

Hydrogen evolution (cathodic reactions):

(6) $$\begin{array}{c} Fe+H^{+}↔{FeH}^{+} \end{array}$$

(7) $$\begin{array}{c} {FeH}^{+}+e^{-}\to FeH \end{array}$$

(8) $$\begin{array}{c} FeH+H^{+}+e^{-}\to Fe+H_{2} \end{array}$$

(9) $$\begin{array}{c} Fe+{Inhibitor}^{+}↔{Fe-Inhibitor}^{+} \end{array}$$

Notwithstanding, the employment of these inhibitors is essentially restricted due to their toxic impacts persisting in the environment and lengthy or costly synthesis approaches [16]. After that, research studies are geared toward exploiting natural sources to develop environment-friendly corrosion amelioration formulations. Green chemistry has predominately attracted attention in the stage of corrosion inhibitor development. Green inhibitors are extracted or synthesized from natural raw materials comprising leaves, seeds, and plant extracts containing parts with expansive properties effective for corrosion mitigation [17]. Despite their negligible toxicity and high biodegradation merits, they exhibit large polymeric sizes with several repeating units, ensuring prominent surface area occupation [18]. Additionally, it is recognized that the existence of some anionic species in the sweet saline solution, which may be attached to the steel surface, induces the protonation of active sites in the polymeric structure with lone pairs of electrons, thereby facilitating the interaction of inhibitor with the surface [19].

Natural or modified biopolymers, specifically gums consisting of long chains of polysaccharides, are an excellent alternative for reducing sweet corrosion spontaneity [[20], [21], [22], [23]]. The gums could be categorized as neutral or ionic, as in the case of guar gum and agar, respectively. Nevertheless, these gums exhibit the drawbacks of uncontrolled hydration rate, solubility dependency on pH, and thermal instability. Integration of gums with surfactants or halide salts is proven to overcome this challenge. As well as chemical grafting, crosslinking, etherification, and esterification are all considered promising modification methods for enhancing anti-corrosion characteristics [24]. The anti-corrosion performance of guar gum grafted methyl methacrylate in 3.5 wt% NaCl saturated with CO_2_ revealed 90% efficiency for P110 steel at 400 mg L^−1^. The study improved it to 96.8% by adding 5 Mm potassium iodide (KI) to a lower GG-MMA concentration of 300 mg L^−1^ [25]. The research group has also investigated the GG grafted with 2-acrylamido-2-methylpropanesulfonic acid and displayed 95% anti-corrosion efficiency of copper at 600 mg L^−1^ in 3.5 wt% NaCl electrolyte at 35 °C [26]. The outstanding performance of two chitosan derivatives in CO_2_-saturated 3.5 wt% NaCl was elucidated by Cui et al. [20], reaching 91 and 93% inhibition efficiencies for corrosion of P110 steel at 100 ppm concentration at 80 °C.

Agar is a heteropolysaccharide composed of agarose (70%) and agaropectin, known to exhibit anti-corrosion properties ascribed to the abundance of electron donor sites in its chemical structure [27]. Literature has archived a substantial role of agar in the corrosion mitigation of aluminum and copper-aluminum alloys [28]. Agar achieved an efficiency of 74.5% for the corrosion inhibition of aluminum alloy at 10 g L^−1^ at 22 °C [29]. Also, it was demonstrated that the 10,000 ppm agar additives reduced the hydrogen evolution from 0.34 ml cm^2^ to 0.12 ml cm^2^ in 4 M NaOH solution in the presence of aluminum [30]. Furthermore, polyacrylamide (PAM), the green biodegradable polymer, has demonstrated efficient inhibitive performance in acidic environments [31]. Subsequently, PAM was integrated with inhibitory chemical chains through graft polymerization reactions to provide formulations with boosted inhibitive characteristics. Graft polymerization with PAM was previously investigated in another corrosion inhibition system, including polyepoxysuccinic acid (PESA) inhibitor [32]. Experimental studies have disclosed the influence of PAM addition on improving efficiency from 43% to 90% at 500 mg L^−1^ concentration. Additionally, in the carbohydrate polymer system, gum acacia-known as the gum arabic (GA)- was grafted with polyacrylamide (AG-PAM) and studied in a 15% HCl solution [33]. The research reported a considerable inhibition efficiency increase of mild steel corrosion from 70% to 90% upon adding 3 g L^−1^ PAM to the GA formulation. Yet, the contribution of PAM grafting to heteropolysaccharide system for corrosion prevention in saline media is not sufficiently reported, and specifically agar investigation in literature is not excessively notable.

The prominent performance of polysaccharides for corrosion inhibition with the possible improvement through graft polymerization has motivated the exploration of novel structures. The biodegradable PAM, known for its efficiency in several corrosive environments, was chosen for grafting to develop a high-performing inhibitor capable of resolving technical problems in the industrial sector and reducing the economic threats related to shutdown and operating costs [34]. In this present research, the anti-corrosion merits of agar and PAM are exploited by developing eco-friendly and bio-tolerable agar-grafted-PAM (AGGPAM) inhibitor for sustainable corrosion prevention. The structure of developed AGGPAM is reported for the first time to testing its anti-corrosion performance for C-steel in highly saline 3.5 wt% NaCl solution saturated with CO_2_, mimicking oilfield conditions. After assessing the toxicity of the synthesized structure, the experimental investigation of AGGPAM comprised electrochemical impedance spectroscopy (EIS) and potentiodynamic polarization (PDP) techniques at various concentrations and temperatures. Nevertheless, the influence of AGGPAM coating on the protection of tested steel was also evaluated. Most importantly, the response of C-steel in blank and inhibited electrolytes was inspected over an extended time at the potentiostatic condition. Lastly, scanning electron microscopy (SEM), energy dispersive X-ray (EDX), and contact angle measurement tests were performed to predict the C-steel surface characteristics prior and post corrosion.

## 2 Materials and methods

### 2.1 Chemicals

Acrylamide (98%), agar gum, and ammonium cerium (IV) nitrate (CAN) (≥99%) were supplied by Glentham Life Science Ltd, United Kingdom. Acetone (99.8%) was supplied by Chem-Lab, Belgium. Deionized water (DI) was used for the preparation of all solutions.

### 2.2 Microwave-assisted synthesis of agar-grafted-polyacrylamide (AGGPAM)

**Fig. 1:**
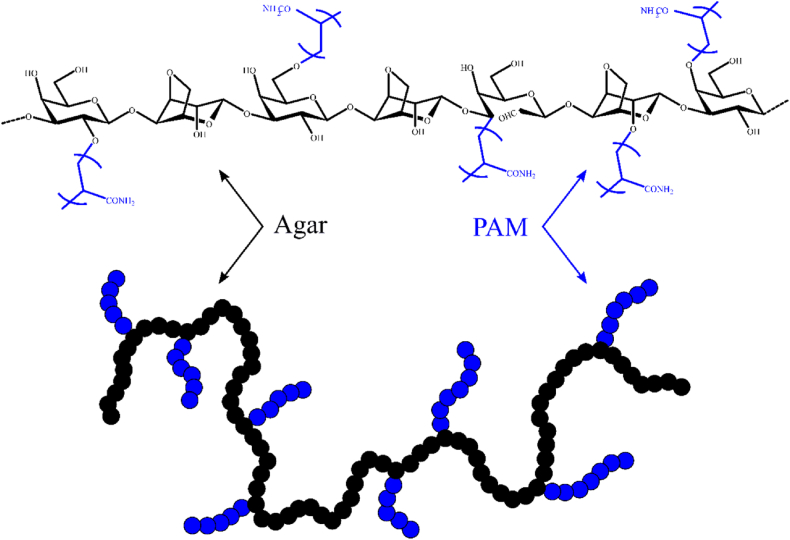
Proposed structure of AGGPAM.

AGGPAM was synthesized according to previous reports with a slight modification [35,36]. Initially, 4 wt% agar solution was prepared, and 0.02 g of cerium ammonium nitrate (CAN) dissolved in 10 ml of DI water was added to the agar solution. The mixture was then stirred and under irradiation of 600 W in a microwave reactor. After 1 min, after the pause of the microwave irradiation, the 10 g of acrylamide solution was added to the mixture. Following that, the irradiation was started again for 5 min and stopped. The reaction vessel was cooled, and the resultant solution became highly viscous, moreover, the grafting reaction could potentially lead to crosslinking [37] and the formation of superabsorbent particles that would swell. As a result, it was a necessary to dilute it to 5% with DI water, filtered, and precipitated in acetone to insure the elimination of undesirable impurities. Finally, the grafted compound (Fig. 1) was dried at 65 °C, crushed and sieved before the collection.

The graft reaction of vinyl monomers onto the agar chain was carried out using a strong redox initiator. Cerium ammonium nitrate can oxidize the polysaccharides ring and produce a free radical. Two types of initiation reactions were proposed in the literature [[38], [39], [40]] (Fig. S1). The first mechanism involves the segregation of the hydrogen atom from the carbon linked to the hydroxyl group, where an intermediate complex C_1_ formed between the ceric ion and the agar. The disproportionation of the complex creates a proton, cerous ion (Ce^3+^), and a free radical on the carbon of agar. The second route was proposed by Usha Rani et al. [35,36], where the nucleophilic groups, such as OH groups, react with ceric ions to form a complex C_2_ and then lead to a free radical on the oxygen of agar. The free radicals zones with the presence of acrylamide monomers can initiate the polymerization to generate graft copolymers.

### 2.3 Steel samples preparation

Carbon steel (C-steel) AISI 1020 alloy sheets were cut into specimens of dimensions 3 × 3 × 0.1 cm. C-steel composition consists of iron and 0.2% carbon with up to 0.7% manganese, 0.65% silicon, and 0.65% copper in wt%. The polishing of steel was done using silicon carbide (SiC) papers for up to a 1500-grit finish. After polishing, deionized water and ethanol were applied to clean the steel. The samples were tested experimentally after drying at a temperature of 110 °C. The steel sample for coating experiments was also prepared following the brush-based layer-by-layer assembly of 500 mg L^−1^ AGGPAM film. Each layer was allowed to dry before the application of the next layer. Lastly, the steel sample was dried at 110 °C for 24 h to ensure a firm sticking of inhibitor film on the surface.

### 2.4 Corrosive solution preparation

The electrolyte solution of the corrosion experiments was a deoxygenated and CO_2_-saturated 3.5 wt% NaCl solution. In order to simulate actual oilfield conditions, the 3.5 wt% NaCl solution was bubbled with N_2_ gas (99.99% purity) for around 30 min. After that, CO_2_ purging was sustained for 1 h to saturate the same solution. At the end of the gas purging, the pH of the solution was adjusted to a pH of 5.0 by adding sodium bicarbonate (NaHCO_3_).

### 2.5 Electrochemical experiments

The design of three-electrode cell was used for the electrochemical tests, and all data was obtained by GAMRY Interface 1600 Potentiostat/Galvanostat/ZRA. The counter and reference electrodes selected for all performed tests are graphite rod and Ag/AgCl electrodes. The working electrode is a 1.0 cm^2^ C-steel sheet exposed to the electrolyte. The experiments were performed using the CO_2_-saturated 3.5 wt% NaCl solution with varying AGGPAM concentrations (0, 50, 100, 200, and 500 mg L^−1^). Continuous CO_2_ bubbling was maintained throughout the entire experiment time. Another set of experiments was performed at 25, 30, 40, and 50 °C in a different jacketed cell, and a Julabo thermostat (GmbH, Seelbach, Germany) was used to adjust the temperatures. The working electrode was initially under a stable open circuit potential (OCP) for around 30 min. Potentiostatic conditions ranging from 100,000 Hz to 0.1 Hz and AC amplitude disturbance of 10 mV were selected for EIS tests. PDP measurements were also obtained at ±250 mV cathodic to anodic potential range against the OCP at an electrode scanning rate of 1.0 mV s^−1^. Each experimental condition was performed in triples to ensure data reproducibility.

### 2.6 Materials characterization

The synthesis of AGGPAM was confirmed by Fourier Transform Infrared Spectroscopic analysis (FTIR) performed using a NICOLET iS10 Thermo Scientific FTIR spectrometer. FTIR spectrum was displayed over a 500-4000 cm^−1^ wavenumber range with a resolution of 16 cm^−1^ and 24 scans.

### 2.7 Surface characterization

The surface of C-steel samples soaked in CO_2_-saturated 3.5%wt NaCl solution with and without 500 mg L^−1^ AGGPAM for 6 h at 25 °C was studied using scanning electron microscopy (SEM) (FE-SEM, FE-SEM-Nova Nano-450, Netherlands) coupled with energy dispersive X-ray (EDX) unit. The characterization was accomplished to compare the surface topography change before and after corrosion.

### 2.8 Eco-toxicity assessment

ADMETlab 2.0 web tool [41] was utilized to assess the eco-toxicity of the synthesized AGGPAM polymer. This tool employs a model formulated from 288,967 experimental entries to assess the absorption, distribution, metabolism, excretion, and toxicity properties. The solubility in water was predicted from the SwissADME website [42], which also adopts a model based on 2874 solubility measurements evaluated versus nine characteristics.

## 3 Results and discussion

### 3.1 FTIR characterization of AGGPAM

**Fig. 2:**
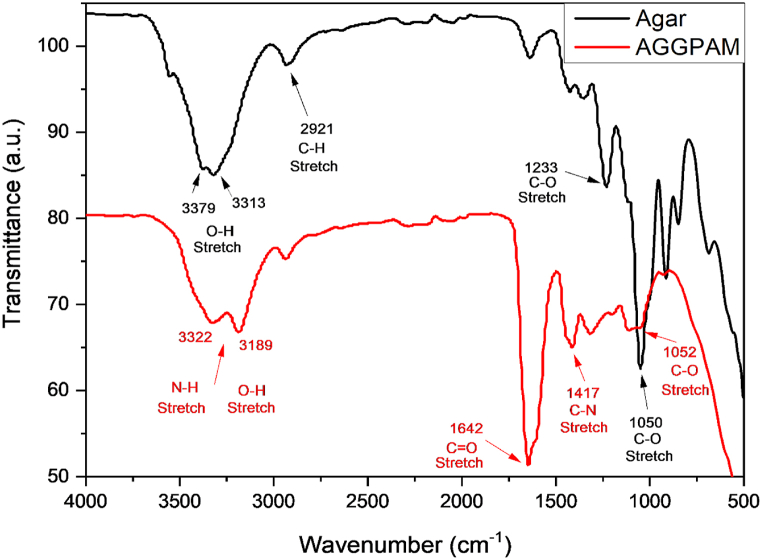
FTIR spectra of Agar and AGGPAM.

The FTIR spectrum of agar in Fig. 2, shows two peaks at 3379 cm^−1^ and 3313 cm^−1^ due to the stretching vibration of OH groups. The peak at 2921 cm^−1^ is assigned to the C–H stretching vibrations and the bands at 1050 cm^−1^ and 1233 cm^−1^ are attributed to the C–O stretching vibrations in ether linkage. In the case of AGGPAM, the spectrum appears with new peaks due to PAM grafting. Peaks at 1642 cm^−1^ and 1417 cm^−1^ are related to the stretching vibrations of C=O and C–N, respectively. The characteristic absorption of N–H, and OH functions appeared in the 3100-3500 cm^−1^ range. The formulation of the grafted structure was proved from the appeared characteristic signs of both agar and PAM, also indicated by the intensity reduction of the agar hydroxyl group in the two peaks at 3379 cm^−1^ and 3313 cm^−1^.

### 3.2 Eco-toxic evaluation

The toxicity assessment of the synthesized AGGPAM polymer shows safe consequences on all considered eco-friendly parameters reported in Table 1. The polymer is considered non-carcinogenic with no harmful impacts on the eyes or the respiratory system. The AGGPAM structure is highly soluble in water, as indicated by the Log (S) value, which must exceed zero to satisfy the criteria for high solubility property.

**Table 1:** Probabilities of eco-toxicity evaluation parameters for AGGPAM synthesized structure.

Property	Probability
*Carcinogenicity*	0.06 (nontoxic)
*Eye Corrosion*	0.003 (nontoxic)
*Eye Irritation*	0.01 (nontoxic)
*Skin Sensitization*	0.082 (nontoxic)
*Respiratory Toxicity*	0.019 (nontoxic)
*Water Solubility,* Log *(S)*	0.47 (highly soluble)

### 3.3 Effect of inhibitor concentration

#### 3.3.1 EIS measurements

Through the corrosion process of C-steel, the species in solution accumulate on the solution-surface interface, creating an electric double layer. The arising double layer has capacitance and resistance for charge transfer. The double layer properties influence the rate of electron transfer between anodic and cathodic zones. EIS measurements elaborate on the change of interface properties, corrosion reaction rate, and surface passivation with the application of an inhibitor [43]. Nyquist and Bode plots for C-steel in the CO_2_-saturated 3.5 wt% NaCl solution with and without different AGGPAM concentrations at 25 °C are illustrated in Fig. 4 (a,b,c). The Nyquist semicircles have a simultaneously increasing diameter with the increase of inhibitor concentration from 50 to 500 mg L^−1^. This suggests enhanced protection and inhibitive performance with the adsorption of many AGGPAM molecules onto the C-steel surface at higher tested concentrations. The dense protective layer at the interface isolates the forthright contact of the C-steel with the NaCl corrosive electrolyte, thus suppressing the rate of metallic dissolution. Comparing the EIS spectra, the diameter for 500 mg L^−1^ inhibited solution is considerably larger than that of the uninhibited medium. It is also evident that the mechanism of charge transfer controls this electrochemical corrosion process, as indicated by the single capacitative loop in all obtained Nyquist plots (Fig. 4 (a)) [44]. The imperfect shape of semicircles can be assigned to the roughness and nonhomogeneity of the steel surface, thereby leading to frequency dispersion.

Bode plots of impedance modulus and phase angle demonstrate highly elevated and broader curves for increased inhibitor concentration conditions (Fig. 4 (b & c)). It is clearly noticed that the absolute impedance increases with incrementing the inhibitor dosage, confirming the role of AGGPAM in creating a protective barrier over the steel surface. The Bode-phase plot in Fig. 4 (c) also elucidates this finding by demonstrating the phase angle increase with the augmentation of inhibitor concentration. The phase angle reached around 55° with the augmentation of inhibitor concentration to 500 mg L^−1^. After all, the single-phase peak indicates a one time constant for the double layer of the studied system [44].

**Fig. 3:**
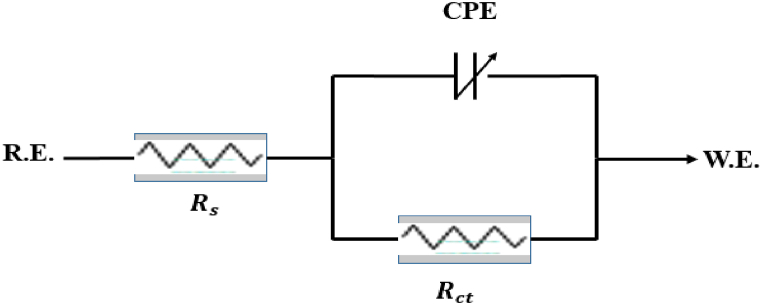
EIS data fitting model.

The equivalent circuit models are exploited to find related parameters of corrosion from the EIS measurements. The components of the fitting model employed in the studied inhibiting system comprise the solution resistance ($R_{s}$), charge transfer resistance ($R_{\text{ct}}$) and constant phase element (CPE), as illustrated in Fig. 3. The obtained EIS parameters are listed in Table 2 with the efficiency of AGGPAM in inhibiting the corrosion of C-steel in the 3.5 wt% corrosive electrolytic medium.

**Fig. 4:**
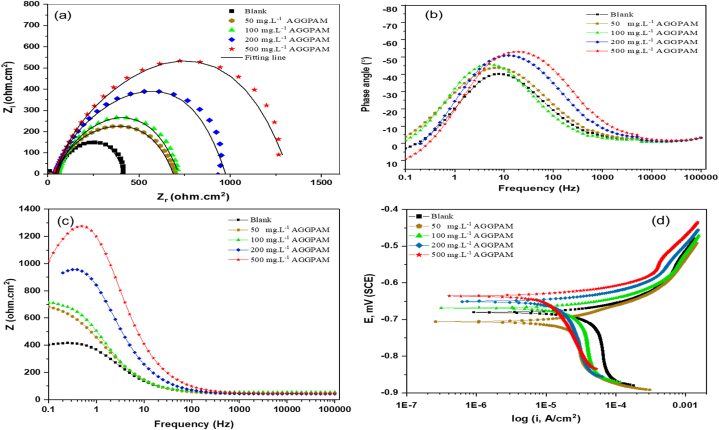
Plots of Nyquist (a), Bode (b,c) and PDP (d) for C-steel in CO2-saturated 3.5% NaCl solution in the uninhibited and inhibited solutions at varying AGGPAM concentrations.

**Table 2:** Effect of AGGPAM concentration on fitted EIS and PDP measurements for C-steel in CO2-saturated 3.5 wt% NaCl.

*EIS Parameters*	C_Inh._ (mg.L^−1^)	R_CT_ (Ω. cm^2^)	R_S_ (Ω. cm^2^)	CPE			IE%
				Y_0_ (μs^n^. Ω^−1^. cm^−2^)	n	$C_{\text{dl}}$ (μF)	
	0	391	45.9	296	0.89	227	**-**
	50	677	42.1	337	0.75	206	42.2
	100	691	55.5	288	0.82	200	43.4
	200	1040	42.1	139	0.79	84.2	62.4
	500	1436	45.0	83.9	0.79	48.1	72.8
*PDP Parameters*	C_Inh._ (mg.L^−1^)	-E_corr_ (mV)	i_corr_ (μA. cm^−2^)	$\beta_{a}$ (mV/decade)	$\beta_{c}$ (mV/decade)	CR (mpy)	IE%
	0	681	72.3	73.2	2.2E-01	33.0	–
	50	697	36.8	88.6	5.8E-02	16.8	49.1
	100	669	33.1	47.4	1.5	15.1	54.2
	200	651	20.6	39.6	1.1	9.4	71.5
	500	636	10.9	27.2	3.3E-01	4.9	84.9

The inhibition efficiency (IE%) can be found by relying on the $R_{\text{ct}}$ values as in equation (10):

(10) $$\begin{array}{c} IE\%=\frac{R_{ct1}-R_{ct2}}{R_{ct1}}\times 100 \end{array}$$

where $R_{\text{ct}1}$ and $R_{\text{ct}2}$ are the charge transfer resistance with and without the inhibitor, respectively.

The electric double-layer capacitance ($C_{\text{dl}}$) is calculated using the following equation (11):

(11) $$\begin{array}{c} C_{dl}=\frac{{\left(Y_{0}R_{ct}\right)}^{1/n}}{R_{ct}} \end{array}$$

The surface inhomogeneity is represented by (n) and is between 0 and 1. $Y_{0}$ is CPE constant. And the thickness of this double layer is related to the capacitance through the following equation (12):

(12) $$\begin{array}{c} C_{dl}=\frac{\varepsilon \;\varepsilon_{0}A}{\delta } \end{array}$$

where $\varepsilon_{0}$ and ε are the dielectric constants for air and water, respectively. A is the cross-sectional area of the electrode.

The CPE impedance is found from the following equation (13):

(13) $$\begin{array}{c} Z_{CPE}=\left(Y_{0}^{-1}{\left(j\omega \right)}^{-n}\right) \end{array}$$

where $Z_{\text{CPE}}$ is CPE impedance in Ω. cm^−2^, $Y_{0}$ is CPE constant in μs^n^. Ω^−1^. cm^−2^, j= (−1)^1/2^ and ω is the angular frequency in rad. s^−1^.

The EIS fitting parameters articulated in Table 2 give evidence of the improved steel resistivity over the varying AGGPAM concentration, which was increased from 391 to 1436 Ω cm^2^. The enhancement of the charge transfer resistance with the AGGPAM could be ascribed to heteroatoms existing in its structure, which strengthens the adsorption onto the steel by donating the π-electrons to Fe atoms. Nevertheless, there is a feasible combination between the positive hydrogen in the solution with the heteroatoms of the inhibitor. The protonated AGGPAM molecules could adsorb onto the negative C-steel surface [45]. This behavior is initiated by the CO_2_ dissolution in the liquid NaCl electrolyte, forming carbonic acid (H_2_CO_3_), which dissociates further to provide more H^+^ as in the chemical reactions in equations (14), (15).

(14) $$\begin{array}{c} CO_{2}+H_{2}O\to H_{2}CO_{3} \end{array}$$

(15) $$\begin{array}{c} H_{2}CO_{3}\to H^{+}+HCO_{3}^{-} \end{array}$$

Additionally, the descending trend of (Y_0_) indicates a homogeneously dispersed AGGPAM film layer, which enhances the compactness of the electric double layer. This could substantially demonstrate the potential motive for the obstructed penetration of corrodent species into the steel surface [46]. The inhibition efficiency reached around 73% at the highest tested AGGPAM concentration. The synthesized AGGPAM inhibitor showed better inhibitive performance than carboxymethyl cellulose (CMC) and chitosan, as studied by Umoren et al. [47] under the similar corrosive environment of the CO_2_-saturated 3.5 wt% NaCl. The $R_{\text{ct}}$ in the presence of CMC and chitosan reached 272 and 332 Ω cm^2^, respectively, which is approximately 50% lower than the acquired for the AGGPAM system. The outstanding performance of AGGPAM could be attributed to the grafting effect of PAM into the agar chain, providing more adsorption sites. Notwithstanding, the nature of PAM polymer –with many repeating units-is another fundamental reason for a superior spreading over the C-steel surface, thus improving the inhibitive effect [32]. Overall, those investigated polysaccharides of CMC and chitosan achieved only 39 and 45% efficiency, proving the impact of PAM grafting.

#### 3.3.2 PDP measurements

Examining potentiodynamic polarization plots illustrates the adsorption mechanism and further elaborates on the anodic and cathodic reactions kinetics. The PDP curves of C-steel in CO2-saturated 3.5 wt% NaCl in the addition of varying AGGPAM concentrations at 25 °C are displayed in Fig. 4 (d). The shift of plots toward increased positive potential values and lower current densities with the increased AGGPAM concentration is clearly observed. Consequently, these plots elucidate the ability of AGGPAM to hinder both the metallic dissolution at the anode and hydrogen evolution at the cathode. The hydrogen evolution is the predominant cathodic reaction, owing to having the cathodic branches with no displayed current plateau. The presence of a current plateau is always an indication of the cathodic diffusion-controlled response [48].

It is worth mentioning that the reaction mechanism is preserved after adding the AGGPAM, as indicated by the plots shape trend at all tested concentrations [49]. However, it can be seen that the anodic curve of the plot with high AGGPAM concentration has exhibited a shoulder trend. This indicates the evolution of Fe-AGGPAM complexes, rearranging and altering the distribution of inhibitor molecules over the surface, leading to enhanced adsorption [48,50]. Literature studies [51] proposed the mechanism and reported that the water molecules are exchanged with inhibitor molecules on the iron surface, as in the reaction shown in equations (16), (17), (18). Thereafter, and during the metallic dissolution, Fe^2+^ forms complexes with the inhibitor settling on the surface. Broad and more evident shoulder at 500 mg L^−1^ is proof of strong chemical bonding and more adsorbents, thereby greater surface coverage. Nevertheless, the shift in cathodic branches is larger with steep slopes, indicating the strong influence of the AGGPAM inhibitor to block the hydrogen evolution reaction.

(16) $$\begin{array}{c} {Inhibitor}_{solution}+{H_{2}O}_{adsorbed}\to \;{Inhibitor}_{adsorbed}+{H_{2}O}_{solution} \end{array}$$

(17) $$\begin{array}{c} Fe\to \;{Fe}^{2+}+e^{-} \end{array}$$

(18) $$\begin{array}{c} {Fe}_{aqueous}^{2+}+{Inhibitor}_{adsorbed}\to \;{Fe\left(Inhibitor\right)}_{adsorbed}^{2+} \end{array}$$

The corrosion electrochemical parameters are estimated from those PDP plots relying on the Tafel extrapolation method. The acquired values of free potential ($E_{\text{corr}}$), current density ($i_{\text{corr}}$), corrosion rate (CR), slopes of anodic ($\beta_{a}$) and cathodic ($\beta_{c}$) branches are all listed in Table 2. The inhibitor efficiency can be calculated using the current density values as in the following equation (19):

(19) $$\begin{array}{c} IE\%=\frac{i_{corr1}-i_{corr2}}{i_{corr1}}\times 100 \end{array}$$

where $i_{\text{corr}1}$ and $i_{\text{corr}2}$ are the current densities of corrosion without and with the inhibitor, respectively.

The obtained parameters demonstrate the influence of increasing the inhibitor concentration on decreasing the current density, thereby diminishing the corrosion rate. When C-steel is exposed to the blank NaCl solution, the corrosion rate is estimated to reach 33 mils per year. However, the gradual addition of AGGPAM into the solution has considerably decreased the corrosion rate until it reached around 5 mils per year. This reduction in the corrosion rate indicates an approximately 85% efficiency achieved at 500 mg L^−1^ AGGPAM. It is inferred that the elevation in AGGPAM concentration minimizes the dissolution of iron. The protective role of AGGPAM in creating a barrier on the C-steel surface can be disclosed from the reduced corrosion rates. Additionally, the corrosion current density values are lowered from 72 for the blank to 11 μA. cm^−2^ for 500 mg L^−1^ AGGPAM, revealing the role of adsorbed inhibitor molecules over the C-steel surface in suppressing the corrosion attacks. The potential is moved to more positive values upon adding more AGGPAM molecules; the values are increased from −697 to −636 mV versus the Ag/AgCl reference electrode. Indeed, the data classify AGGPAM as a mixed-type inhibitor since the polarization potential does not exceed ±85 mV [52]. Subsequently, this also implies that AGGPAM has inhibitory functions on both anodic dissolution of steel and cathodic evolution of hydrogen.

The synthesized AGGPAM has proved to exceed the inhibition performance of chitosan, and CMC tested in the same corrosive conditions, which reported around 55% as per the potentiodynamic experiments [47]. Furthermore, Roy et al. [53] reported that grafting PAM on guar gum attained higher anti-corrosion efficiency, reaching 90% at 500 mg L^−1^ with 7.5% gum percentage in the grafted composite. The corrosion current density was remarkably diminished from 550 μA. cm^−2^ in blank 1.0 M HCl to 130 μA. cm^−2^ at this testing concentration. Gum acacia (GA) grafted with PAM showed a remarkable current density reduction from 1053 μA. cm^−2^ for mild steel in blank 15% HCl to 86.76 μA. cm^−2^ in inhibited solution with 200 ppm GA-g-3PAM [33]. This suggests that PAM grafting has facilitated the availability of more –CONH_2_ and –OH groups in the inhibitor structure, providing easier electron transfer and stronger adsorption onto the surface [54].

### 3.4 Effect of temperature

**Fig. 5:**
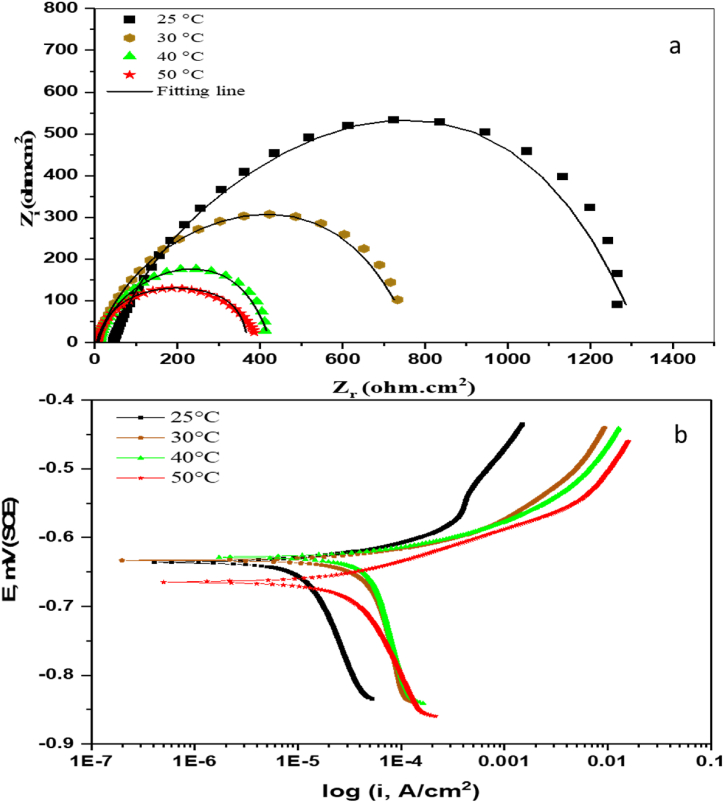
The impact of temperature on (a) EIS and (b) PDP measurements of C-steel in CO_2_-saturated 3.5% NaCl solution and with different AGGPAM concentrations.

The impact of varying temperatures on C-steel corrosion in the CO_2_-saturated 3.5 wt% NaCl was explored at the optimum tested concentration of 500 mg L^−1^. The EIS and PDP plots at 25, 30, 40, and 50 °C are illustrated in Fig. 5. The impedance curves in Fig. 5 (a) elucidate sequentially depressed semicircles with the temperature elevation, representing a declined protection against corrosion in this saline environment. The variations in thermal conditions revealed a conserved corrosion mechanism, as indicated by the maintained shape of all semicircles. However, the smaller diameter of semicircles indicates accelerated electrochemical reactions and metal dissolution, thereby increasing the corrosion at high temperatures [55].

Furthermore, the PDP curves in Fig. 5 (b) demonstrate the gradual shift to more negative cathodic and anodic polarization potentials and higher current densities with temperature rise. These noticeable shifts are translated into deteriorated inhibition performance of AGGPAM at higher temperatures. Nevertheless, the cathodic branches confirm that the cathodic reaction is controlled by hydrogen evolution, as there were no current plateaus. This also indicates that temperature has not changed the corrosion mechanism with the existence of AGGPAM under the tested condition.

The fitted electrochemical measurements performed at various temperatures are listed in Table 3. Firstly, a significant deterioration in the charge transfer resistance is noticed, declining from 1436 to 355 Ω cm^2^ over the overall temperature rise of 25 °C. This is also confirmed by increased double-layer capacitance with the temperature, where the lowest capacitance of 48 μF is reported at 25 °C. The negative influence of temperature on corrosion inhibition properties, essentially due to the activity obstruction of chemical inhibitory structures, is widely reported in the literature [56]. The system exhibits an equilibrium imbalance between the adsorption and desorption rate upon the temperature increase [57]. The desorption of AGGPAM is induced at higher temperatures, thus leaving a greater surface area susceptible to the access of aggressive corrosive species. EIS showed an inclusive performance deterioration for AGGPAM of around 75%, relying on the steel resistivity. This finding is also supported by the increased values of $Y_{0}$ at elevated temperatures, indicating reduced compactness of the inhibitory film owing to the weakly adsorbed AGGPAM molecules [46]. At the highest tested temperature of 50 °C, the $Y_{0}$ is raised to 176 μs^n^. Ω^−1^. cm^−2^ compared to 83.9 μs^n^. Ω^−1^. cm^−2^ at 25 °C.

**Table 3:** Temperature effect on fitting of EIS and PDP measurements for C-steel in CO_2_-saturated 3.5 wt% NaCl.

*EIS Parameters*	T (°C)	R_CT_ (Ω. cm^2^)	R_S_ (Ω. cm^2^)	CPE		
				Y_0_ (μs^n^. Ω^−1^. cm^−2^)	n	$C_{\text{dl}}$ (μF)
	25	1436	45.0	83.9	0.79	48.1
	30	822	4.9	226	0.81	151
	40	434	8.4	196	0.85	126
	50	355	5.5	176	0.82	94.2

*PDP Parameters*	T (°C)	-E_corr_ (mV)	i_corr_ (μA. cm^−2^)	$\beta_{a}$ (mV/decade)	$\beta_{c}$ (mV/decade)	CR (mpy)
	25	636	10.9	27.2	330	4.9
	30	633	19.1	31.8	322	17.4
	40	628	24.5	30.2	855	22.4
	50	665	29.2	75.1	613	26.7

It is worth stating that another main cause for the decremented corrosion protection could be the enhanced aggressive electrolyte diffusion at higher temperatures. Indeed, the corrosion phenomenon is initiated by the diffusion of electrolytes and the ingress of the corrosive ions and oxygen [58]. To further explain this, the kinetics of metallic oxidation comprise either outward diffusion of cations through oxides or inward diffusion of oxygen [59]. The weakly adsorbed AGGPAM on the steel surface results from the imbalance between adsorption/desorption kinetics at high temperatures, promoting electrolyte ingress and oxygen diffusion through the pores [60]. All these influential factors combined elucidate an accelerated corrosion rate from 4.9 to 26.7 mils per year at this temperature change from 25 to 50 °C in 500 mg L^−1^ AGGPAM inhibited solution. Likewise, the current density is also incremented by 167% from 10.9 to 29.2 μA cm^−2^ over the inspected temperature range. The potential of corrosion is moved to increased negative values from −636 to −665 mV upon the temperature increase. This behavior at high temperatures is usually associated with the evolution of surface roughness, affecting the inhibitor molecules to favor desorption [61]. All things considered, the AGGPAM molecules exhibit a diminished capability to create a protective barrier over the C-steel surface at elevated temperatures. Nevertheless, the synthesized AGGPAM demonstrated superior performance to another natural polysaccharide of guar gum grafted methyl methacrylate at 50 °C in the same corrosive electrolyte, undergoing a corrosion rate of 105 mils per year [25].

The inhibitor deficiency trend with increasing temperature is expected to result from the weakened physisorption of inhibitor onto the corroding metallic surface [62]. The activation energy can be estimated by correlating the temperature impact on corrosion kinetics through the Arrhenius equation (20).

(20) $$\begin{array}{c} \log\;\left(CR\right)=\frac{-E_{a}}{2.303\;R\;T} \end{array}$$

where CR is the corrosion rate, $E_{a}$ is the activation energy, R is the universal gas constant in J.mol^−1^. K^−1^ and T is the temperature in K.

Consequently, the change of other thermodynamic parameters can be estimated from the Arrhenius transition-state equation (21) relying on the corrosion rate as [63,64]:

(21) $$\begin{array}{c} CR=\frac{R\;T}{N\;h}e^{\frac{\Delta S^{*}}{R}}e^{\frac{-\Delta H^{*}}{R\;T}} \end{array}$$

where CR is the corrosion rate, R is the universal gas constant in J.mol^−1^. K^−1^, T is the temperature in K, N is the Avogadro number, h is the Planck constant, ${\Delta H}_{a}$ is the activation enthalpy in kJ. mol^−1^ and ${\Delta S}_{a}$ is the activation entropy in J. mol^−1^. K^−1^.

**Fig. 6:**
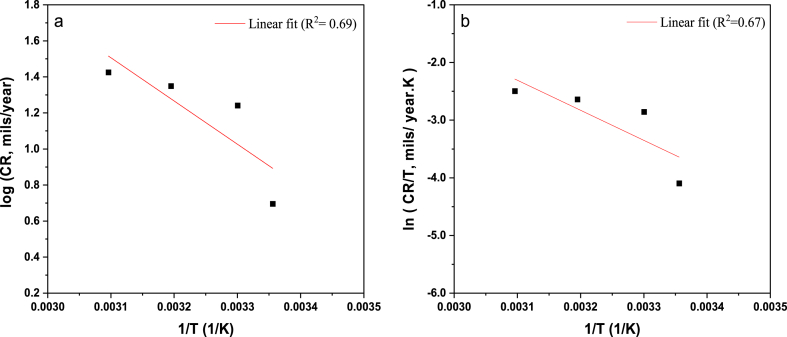
Plots of (a) Arrhenius and (b) transition-state for C-steel in CO_2_-saturated 3.5 wt% NaCl with 500 mg L^−1^ AGGPAM.

The apparent activation energy is estimated at the value of 45.9 kJ mol^−1^ from the slope of Fig. 6 (a), indicating a physical nature of AGGPAM adsorption into the C-steel surface since it does not exceed the chemisorption threshold of 80 kJ mol^−1^ [65]. Additionally, the slope and intercept of Fig. 6 (b) are used to determine the enthalpy and entropy of corrosion.

Calculations revealed 43 kJ mol^−1^ and 115 J mol^−1^ for ${\Delta H}^{*}$ and ${\Delta S}^{*}$, respectively. The positive enthalpy interprets an endothermic corrosion process, where energy is adsorbed during the metal dissolution. It is suggested that the inhibited solution exhibits energy adsorption due to the complexes composed between the inhibitor and the iron atoms [66]. Moreover, the positive entropy value denotes high disorderliness with unstable metal dissolution and hydrogen-induced cracking. Indeed, the temperature induces the formation of activated complexes, thus growing the randomness. The thermodynamic parameters disclose an unimolecular corrosion phenomenon with AGGPAM since the difference of $E_{a}-{\Delta H}_{a}$ is nearly equivalent to the RT value [67].

### 3.5 Investigation of AGGPAM coating film

**Fig. 7:**
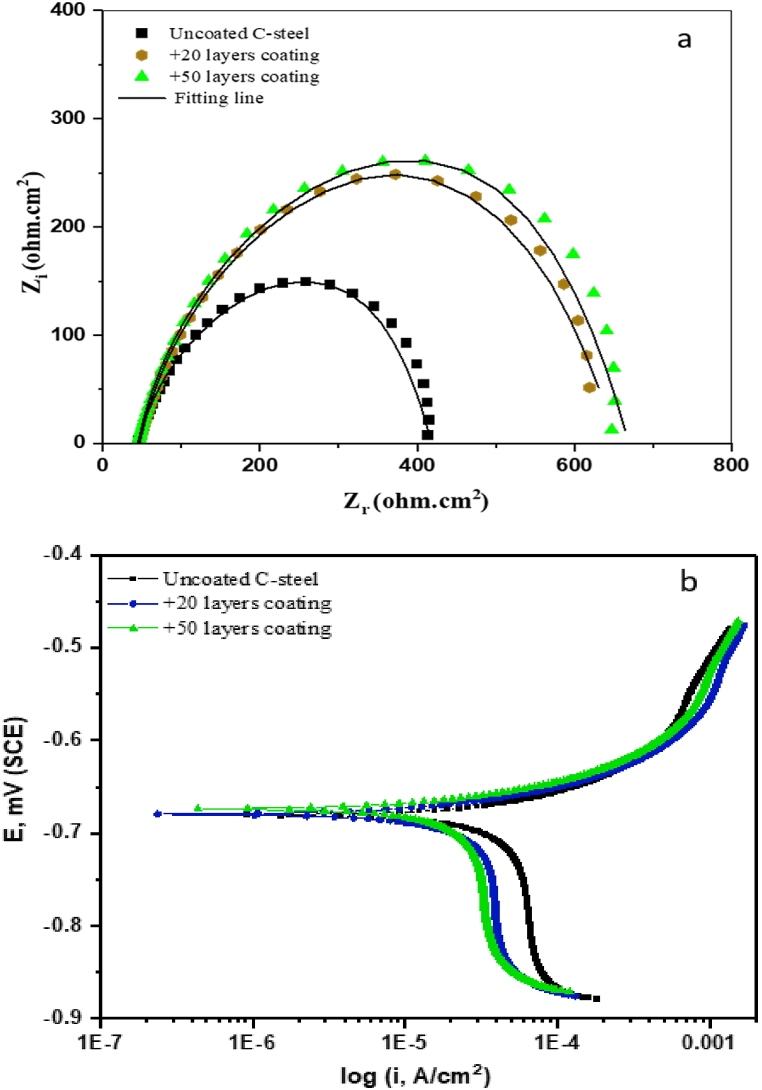
AGGPAM coating effect on (a) EIS and (b) PDP measurements for corrosion of C-steel in CO_2_-saturated 3.5 wt% NaCl at 25 °C.

The anti-corrosion behavior of AGGPAM coating was also inspected through EIS and PDP electrochemical techniques. Fig. 7 compares the electrochemical measurements of uncoated C-steel with two different applied coating layers of AGGPAM in CO_2_-saturated 3.5 wt% NaCl at 25 °C. The effect of low and high surface coverage is evaluated by assessing the two different coated steel specimens with varying AGGPAM layers. Firstly, the enlarged diameters of Nyquist semicircles (Fig. 7 a) demonstrate the role of coating layers in improving the steel resistance of charge transfer with 20 and 50 AGGPAM layers. The identical shape of Nyquist plots affirms maintaining the exact corrosion mechanism exhibited by the uncoated C-steel. A moderate shift of PDP cathodic branches to less negative potentials and reduced current densities is observed (Fig. 7 b), while no significant anodic change is noticed. However, the corrosion potential shift is lower than 85 mV, classifying it again as a mixed-type inhibitor. Therefore, it is evident that these techniques have explained the influence of AGGPAM layers over the C-steel surface, behaving as a corrosion preventive barrier.

The performance of the obtained coating is initially assessed from the inhibition efficiency of fitted EIS and PDP curves. Outcomes admit the initial success of this developed coating film in achieving up to 71% corrosion inhibition by reducing the corrosion rate from 33 to 9.6 mils per year, as in Table 4. This suggests that enhancing this coating film through flow or dip coating techniques could further improve the protection performance [68]. Moreover, the lower value of Y_0_ for the coated C-steel surface indicates an extra compact film on the coated C-steel surface rather than the corrosion product film on the uncoated steel surface [69]. The charge transfer resistance is enhanced by around 73% from 391 to 677 Ω cm^2^, emphasizing the favorable inhibition effect of the AGGPAM coating film.

**Table 4:** AGGPAM coating effect on EIS and PDP measurements for C-steel corrosion in CO2-saturated 3.5 wt% NaCl at 25 °C.

*EIS Parameters*	Layers	R_CT_ (Ω. cm^2^)	R_S_ (Ω. cm^2^)	CPE			IE %
				Y_0_ (μs^n^. Ω^−1^. cm^−2^)	n	$C_{\text{dl}}$ (μF)	
	0	391	45.9	296	0.89	227	–
	20	622	47.5	278	0.82	187	37.2
	50	677	45.0	206	0.82	132	42.3
	
*PDP Parameters*	Layers	-E_corr_ (mV)	i_corr_ (μA. cm^−2^)	$\beta_{a}$ (mV/decade)	$\beta_{c}$ (mV/decade)	CR (mpy)	IE %
	0	681	72.3	73.2	222	33.0	–
	20	680	23.7	43.7	319	10.8	67.2
	50	674	21.0	38.0	358	9.6	71.0

### 3.6 Potentiostatic experiment

**Fig. 8:**
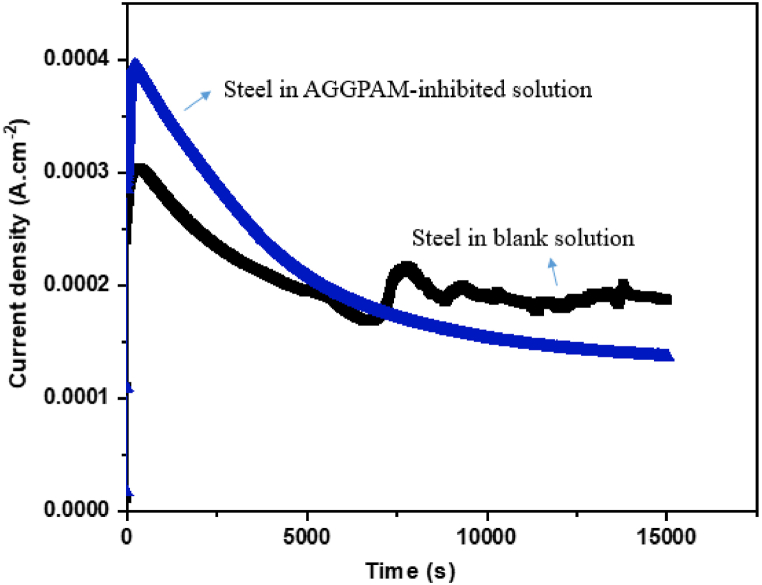
Potentiostatic experiment at 50 mV above the E_corr_ for C-steel in CO_2_-saturated 3.5 wt% NaCl for the uninhibited and AGGPAM-inhibited solutions.

A potentiostatic experiment is performed using the potentiostatic technique to evaluate the corrosion response of C-steel over a prolonged period of 4 h in steady-state conditions at initial and final potentials of 0 and 50 mV, respectively [70]. Fig. 8 illustrates the current density versus time for C-steel corrosion in CO_2_-saturated 3.5 wt% NaCl at an applied 50 mV above the corrosion potential for 4 h with and without 500 mg L^−1^ AGGPAM in the corrosive electrolyte. At the start of the experiment, the current density increases as the polymer starts to be adsorbed and partially covers the surface, thus destabilizing the surface potential and reacting with the iron ions on the surface to be incorporated through surface dissolution/bonding. With time passing, the film stabilizes and spreads over the whole surface, leading to a decreased current response.

It is noticed that the blank curve exhibited an increase in the current density response after around 2 h to an approximate average current of 200 μA cm^−2^, with insignificant fluctuations. In contrast, the response curve of the C-steel in the AGGPAM-inhibited electrolyte shows a decay to lower current density values with time, reaching around 137 μA cm^−2^. It is worth clarifying that the shift to more positive current values could represent the start of steel surface breakdown in the blank saline electrolyte. Nevertheless, the metallic surface is protected when exposed to inhibited solution as indicated by the lower current density, ascribed to developing a thick protective barrier against corrosion [71].

### 3.7 Contact angle measurements

The hydrophobic and hydrophilic alteration of the steel surface was predicted from contact angle measurements to analyze the response after the inhibitor adsorption. Fig. S2 displays the cross-sectional images of a water droplet with 10-μL volume deposited above the C-steel surface immersed in the corrosive solution with and without 500 mg L^−1^ AGGPAM for 6 h at 25 °C. The average contact angle was considered after collecting around ten measurements at different points. It is distinctly shown that the average contact angle increased from 72.4° to 83.2° with standard deviations of 0.3 and 0.7, respectively, when AGGPAM was added to the corrosive electrolyte. The reported outcomes underline the impact of inhibitor film, rendering the steel more hydrophobic, thereby, the long chains of agar polysaccharide with polymeric PAM expelling water molecules from the metal/electrolyte interface [72]. Overall, it is inferred that AGGPAM has a compatible potential to form a protective anti-corrosion film.

### 3.8 Morphological analyses

#### 3.8.1 SEM

**Fig. 9:**
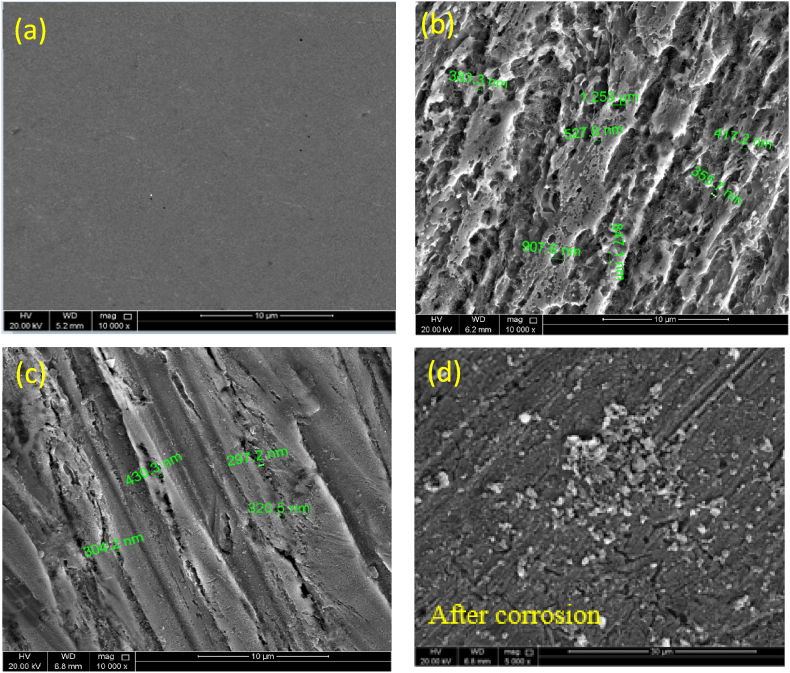
SEM micrographs of (a) polished C-steel samples in (b) blank CO2-saturated 3.5 wt% NaCl (c) with 500 mg.L-1 AGGPAM inhibitor immersed for 6 h, and (d) AGGPAM coated steel sample before and after 6 h immersion, all at 25 °C.

SEM analysis is performed to obtain profound insights into the corrosion mechanism with the addition of the synthesized polysaccharide-based inhibitor. SEM micrographs of the C-steel surface prior and post corrosion occurred from the submerging of samples in blank CO_2_-saturated NaCl solution with and without 500 mg L^−1^ AGGPAM inhibitor are presented in Fig. 9 (more images are in Fig. S3). The topography of polished C-steel (Fig. 9 a) **shows** a flat smooth surface without defects, yet scratches resulted from the polishing stage. In contrast, immersion of steel sample in the CO_2_-saturated 3.5 wt% NaCl electrolyte has significantly deteriorated the surface, where deep pits and cracks have emerged (Fig. 9 b). This indicates the detrimental influence of the corrosive electrolyte on the destruction of the C-steel surface. Nevertheless, the protective role of AGGPAM in forming an efficient shielding layer over the surface is inferred from the improved topography in (Fig. 9 c), where the addition of AGGPAM diminished the roughness of steel and reduced the volume and number of holes. The size of the pits measured for C-steel in the blank corrosive saline solution was approximately 350 nm to 1.3 μm. The steel pits in the AGGPAM-inhibited highly saline solution were small to 290 nm and the largest to 430 nm. This also assures enhanced protection upon adding AGGPAM, behaving as an efficient corrosion inhibitor [19]. Furthermore, the topography alteration of the developed AGGPAM coating discussed above is investigated, as in (Fig. 9 d). The SEM image of coated steel demonstrates surface differences compared to the ordinarily polished steel surface, where there seems to be a layer spreading over the surface. The two images of the coating after corrosion experiments illustrate the distribution of polymeric substances aggregations with corrosion products onto the surface. Herein, the presence of AGGPAM coating is proved, representing the improved surface conditions and diminished damages [73]**.**

#### **3.8.2** EDX

**Fig. 10:**
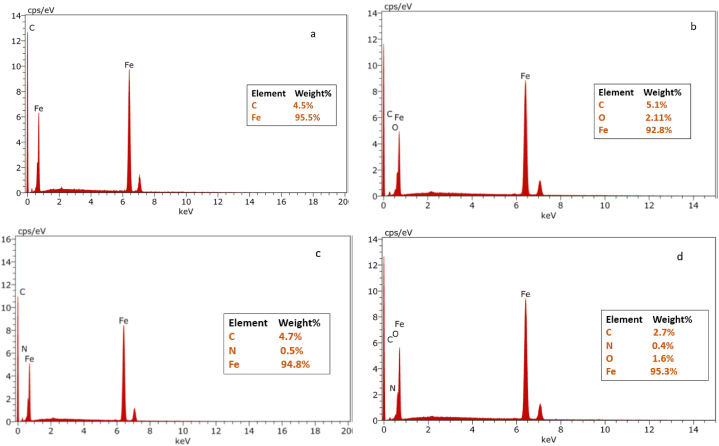
EDX spectra of (a) polished C-steel in (b) blank CO_2_-saturated 3.5 wt% NaCl with (c) 500 mg L^−1^ AGGPAM inhibitor or (d) with AGGPAM coating before and after 6 h of immersion at 25 °C.

The EDX analysis is performed to predict the existing chemical components above the C-steel surface with and without AGGPAM inhibitor in electrolyte and as a coating film. Fig. 10 displays the EDX spectra of polished C-steel (Fig. 10 (a)) immersed in uninhibited (Fig. 10 (b)) and inhibited highly saline electrolytes (Fig. 10 (c)) alongside the AGGPAM-coated C-steel specimen (Fig. 10 (d)). The detected oxygen percent in Fig. 10 (b) indicates a corrosion exhibited by the steel specimen. The oxygen detection is proof of the built iron oxide layer above the corroded surface. Notwithstanding, the steel immersed in inhibited solution Fig. 10 (c) reveals a weight% of nitrogen, ascribed to the presence of adsorbed AGGPAM molecules. The nitrogen existence is known to be sourced from the PAM polymer chain. This EDX study also affirms the presence of nitrogen in the AGGPAM-coated steel specimen Fig. 10 (d). It is worth reporting that oxygen is not detected in the specimen soaked in the AGGPAM-inhibited solution (Fig. 10 (c)), revealing the insignificant formation of the oxide layer corresponding to efficient corrosion protection. Ultimately, the surface characterization study provided trustworthy outcomes complying with the electrochemical assessment.

## 4 Conclusions

The present research study focuses on synthesizing a biodegradable environment-friendly corrosion inhibitor compound relying on natural agar gum. The inhibitor structure of AGGPAM was explored in a CO_2_-saturated 3.5 wt% NaCl medium for C-steel corrosion. Toxicity evaluation using ADMETlab predicted safe impacts for AGGPAM on all considered ecological parameters. Electrochemical measurement results demonstrated the role of concentration in enhancing the anti-corrosion performance. The efficiency of AGGPAM is proven to reach around 85% at 500 mg L^−1^, where the reported corrosion rate was 4.9 mils per year, confirming the potential of protective barrier formation over the steel surface. The inhibitor performed well at elevated temperature of 50 °C where the corrosion rate was found to be 26.7 mils per year, which is still lower than the rate in the uninhibited solution at 25 °C. The AGGPAM-coated C-steel elucidated the success of the developed film in achieving significant inhibition. A comparative analysis of the current curves at a constant potential of 50 mV above OCP for C-steel in the uninhibited and inhibited NaCl solutions reveals a breakdown of the uninhibited specimen, as noticed from the increase in the current after 2 h, while the current of the inhibited surface kept decreasing even after 4 h reflecting the long term stability of the polymeric film. Lastly, reduced hydrophobicity from contact angle measurements, SEM, and EDX analyses indicated that AGGPAM adsorbs onto the C-steel surface, effectively reducing the corrosion rate. The synthesized AGGPAM composite exhibited a prominent performance in the CO_2_-saturated highly saline solution. Despite the remarkable protection, the AGGPAM efficiency could be further enhanced in future studies when mixed with co-inhibitors under proper compositions for developing superior inhibition formulations.

## Author contribution statement

Rem Jalab; Mazen Khaled: Conceived and designed the experiments; Performed the experiments; Analyzed and interpreted the data; Contributed reagents, materials, analysis tools or data; Wrote the paper. Mohammed Saad: Conceived and designed the experiments; Contributed reagents, materials, analysis tools or data. Ahmed Benali; Ibnelwaleed Hussein: Contributed reagents, materials, analysis tools or data.

## Data availability statement

Data will be made available on request.

## Declaration of competing interest

The authors declare that they have no known competing financial interests or personal relationships that could have appeared to influence the work reported in this paper.
