# Supplementary material for: Biodegradable polysaccharide grafted polyacrylamide inhibitor for corrosion in CO2- saturated saline solution
Source: Heliyon. 2023 Sep 20;9(10):e20304. doi: 10.1016/j.heliyon.2023.e20304 (PMC10556602; doi:10.1016/j.heliyon.2023.e20304)
Supplement: Multimedia component 1 [file mmc1.docx]

**Biodegradable Polysaccharide Grafted Polyacrylamide Inhibitor for Corrosion in CO_2_- Saturated Saline Solution**

**Supplementary Information**

## ***2.2 Microwave-Assisted Synthesis of Agar-Grafted-Polyacrylamide (AGGPAM)***

*
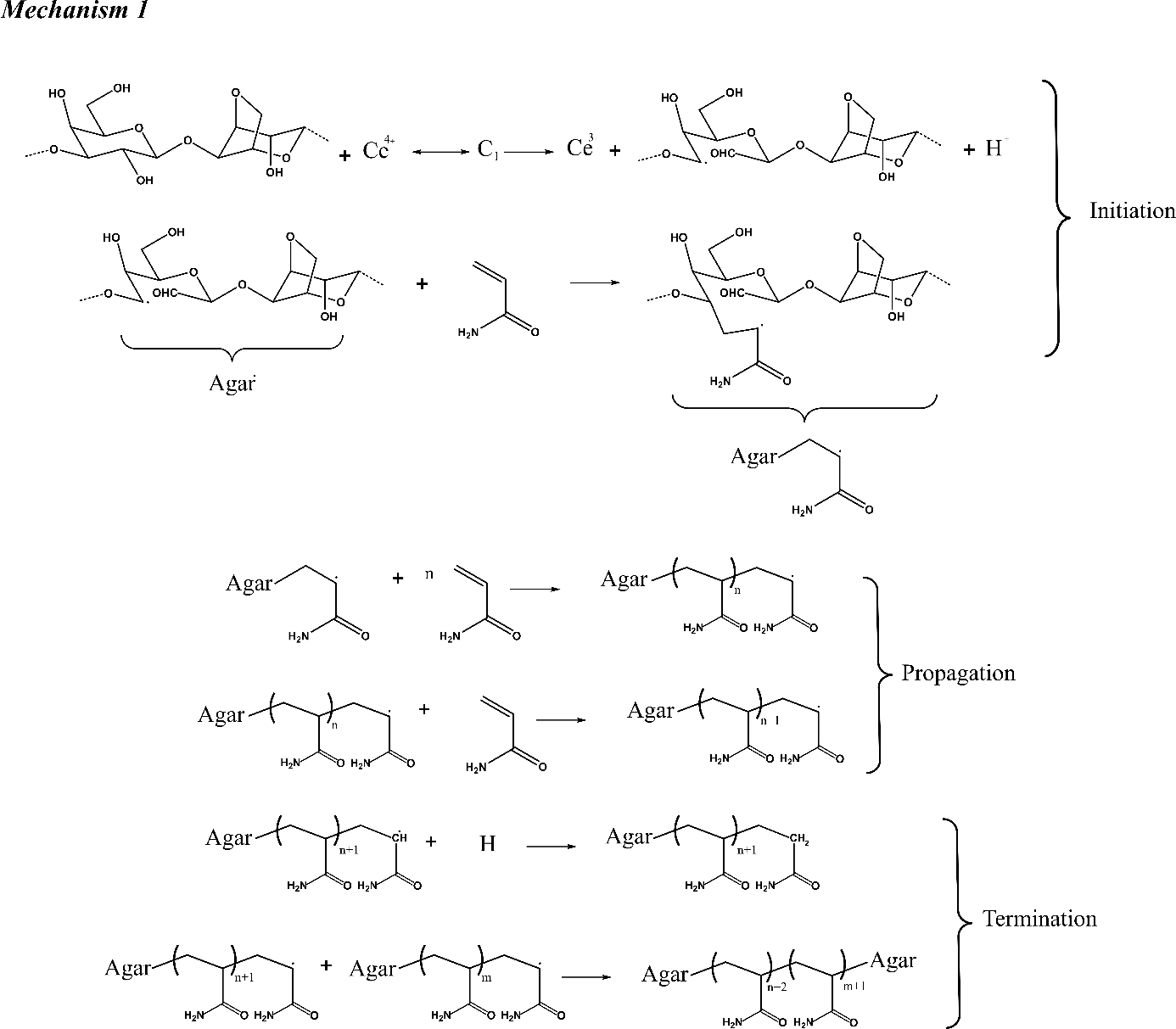
*


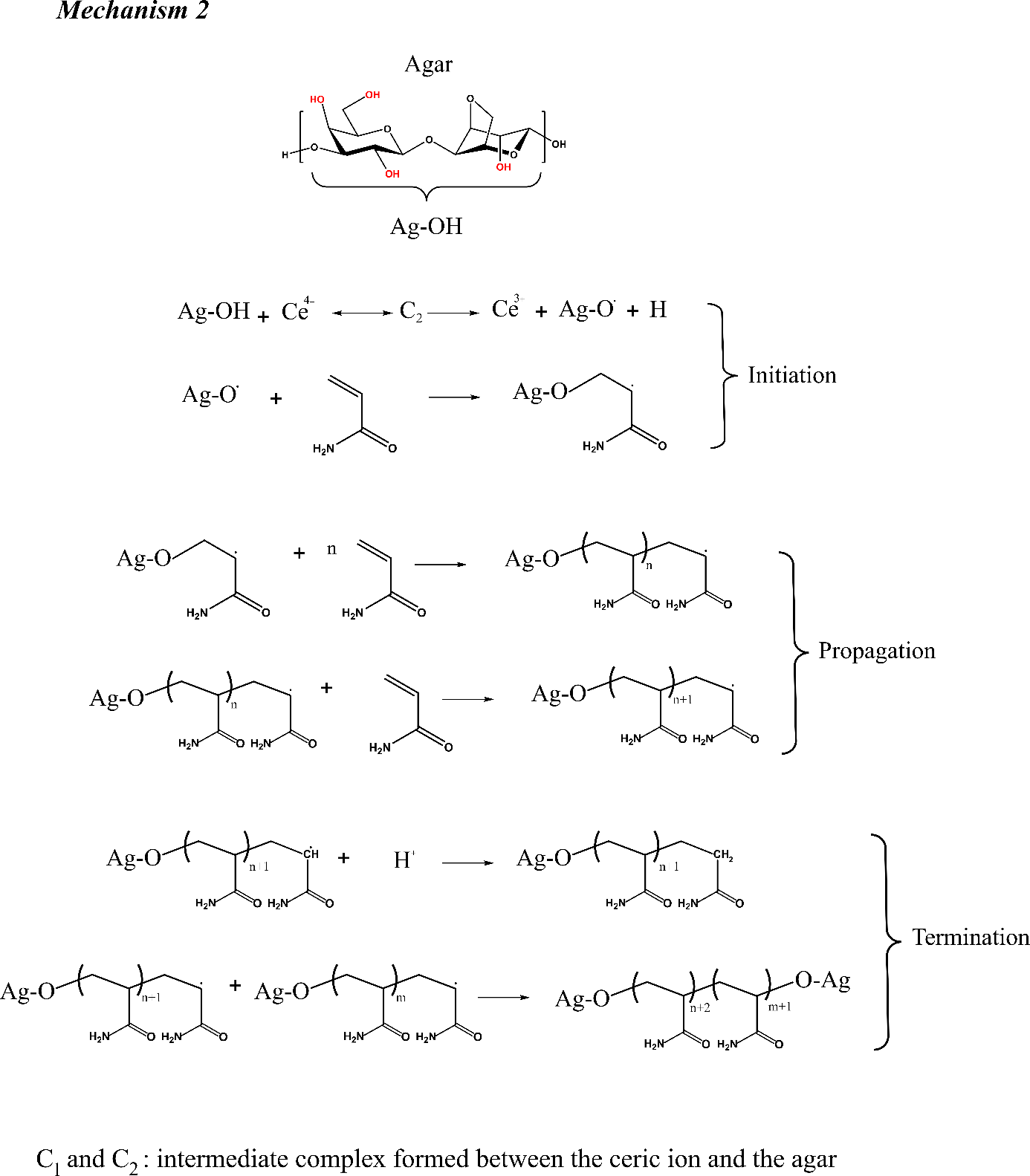


**Figure S1. Proposed reaction mechanisms for PAM graft polymerization onto agar chain.**

## ***Contact Angle Measurements***

| 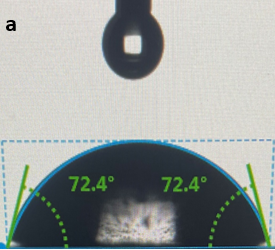 | 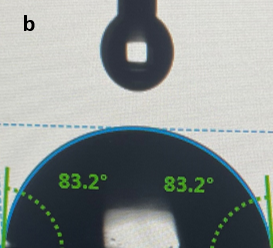 |
| --- | --- |

**Figure S2. Contact angle measurements of C-steel in (a) blank CO_2_-saturated 3.5 wt% NaCl with (b) 500 mg.L^-1^ AGGPAM inhibitor after 6 hours immersion at 25 °C.**

| 1. *Polished C-steel* | 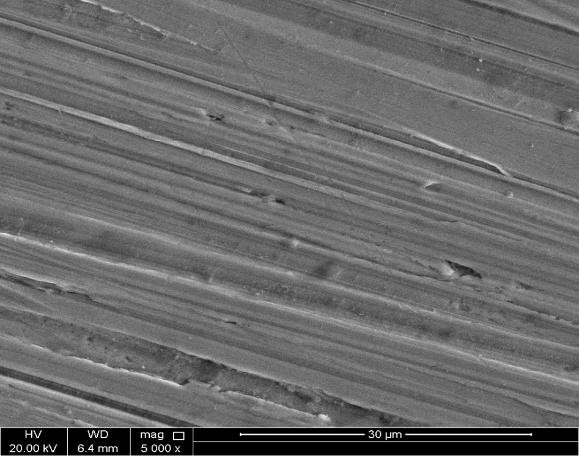 | 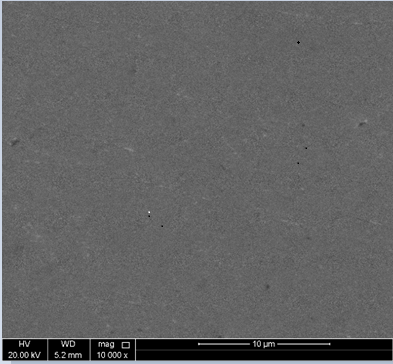 | 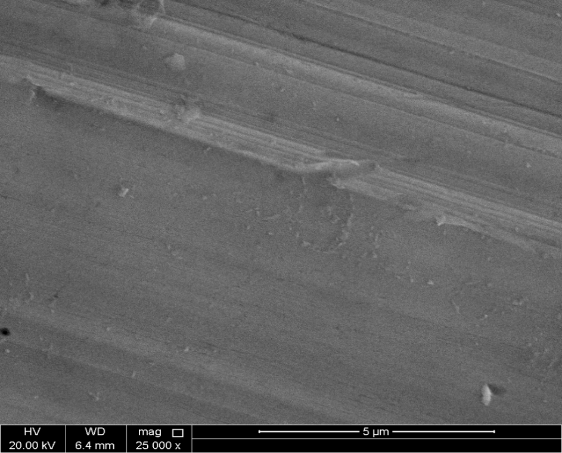 |
| --- | --- | --- | --- |
| 1. *C-steel in blank* | 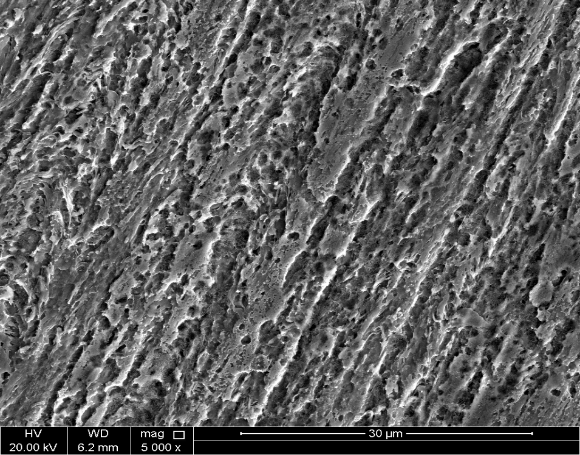 | 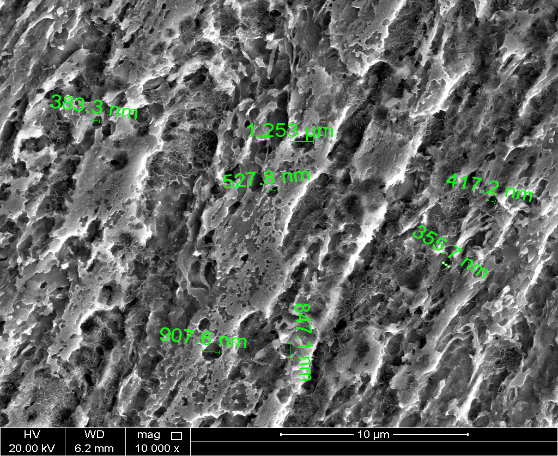 | 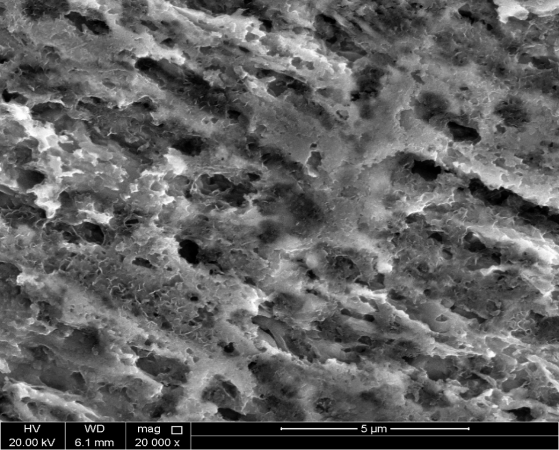 |
| 1. *C-steel in solution with 500 mg.L^-1^ AGGPAM* | 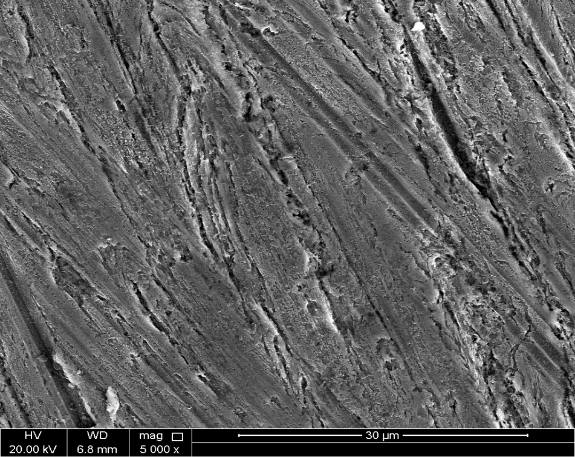 | 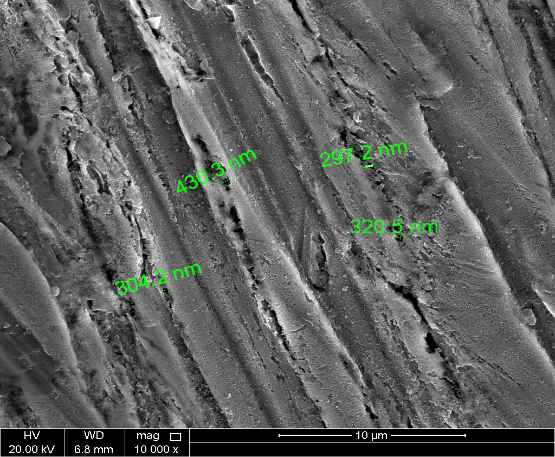 | 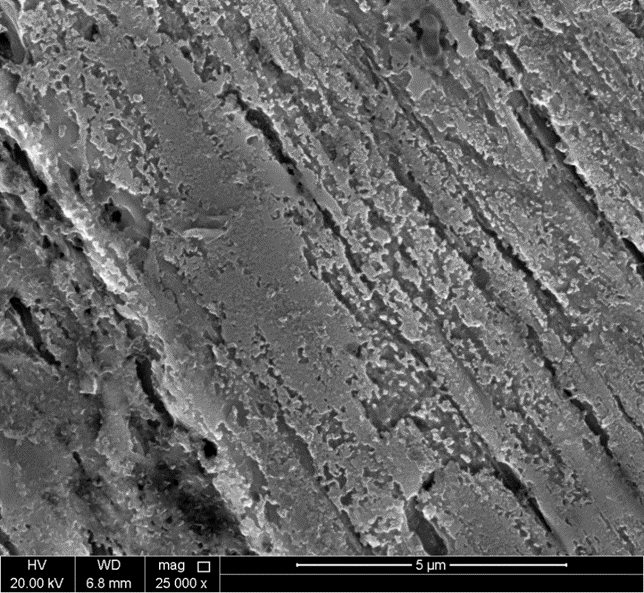 |
| 1. *AGGPAM Coated C-steel* | 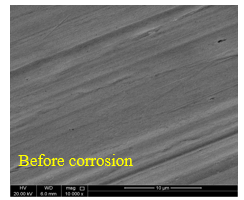 | 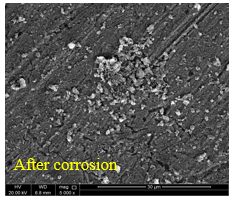 | 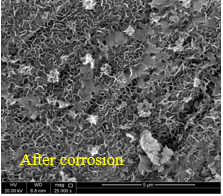 |

**Figure S3. SEM micrographs of (a) polished C-steel samples in (b) blank CO2-saturated 3.5 wt% NaCl (c) with 500 mg.L-1 AGGPAM inhibitor immersed for 6 hours, and (d) AGGPAM coated steel sample before and after 6 hours immersion, all at 25 °C**
